# Splenic Red Pulp Macrophages Produce Type I Interferons as Early Sentinels of Malaria Infection but Are Dispensable for Control

**DOI:** 10.1371/journal.pone.0048126

**Published:** 2012-10-29

**Authors:** Charles C. Kim, Christopher S. Nelson, Emily B. Wilson, Baidong Hou, Anthony L. DeFranco, Joseph L. DeRisi

**Affiliations:** 1 Division of Experimental Medicine, Department of Medicine, University of California San Francisco, San Francisco, California, United States of America; 2 Department of Biochemistry and Biophysics, University of California San Francisco, San Francisco, California, United States of America; 3 Department of Microbiology and Immunology, University of California San Francisco, San Francisco, California, United States of America; 4 Institute of Biophysics, Chinese Academy of Sciences, Beijing, China; 5 Howard Hughes Medical Institute, University of California San Francisco, San Francisco, California, United States of America; Agency for Science, Technology and Research - Singapore Immunology Network, Singapore

## Abstract

Type I interferons (T1IFNs) are among the earliest cytokines produced during infections due to their direct regulation by innate immune signaling pathways. Reports have suggested that T1IFNs are produced during malaria infection, but little is known about the *in vivo* cellular origins of T1IFNs or their role in protection. We have found that in addition to plasmacytoid dendritic cells, splenic red pulp macrophages (RPMs) can generate significant quantities of T1IFNs in response to *P. chabaudi* infection in a TLR9-, MYD88-, and IRF7-dependent manner. Furthermore, T1IFNs regulate expression of interferon-stimulated genes redundantly with Interferon-gamma (IFNG), resulting in redundancy for resistance to experimental malaria infection. Despite their role in sensing and promoting immune responses to infection, we observe that RPMs are dispensable for control of parasitemia. Our results reveal that RPMs are early sentinels of malaria infection, but that effector mechanisms previously attributed to RPMs are not essential for control.

## Introduction

Early recognition of infection by innate immune defenses initiates a complex cascade of intra- and intercellular signaling events that ultimately leads to the generation of a systemic immune response. Although detailed analysis of early innate immune events is under way for model organisms such as *Listeria* [1], relatively little is understood about early detection and responses to *Plasmodium spp.,* the leading parasitic cause of infectious mortality and morbidity in the world. This is despite growing evidence that innate immune responses, particularly of monocytes and macrophages, play a vital role in the control of malaria infection. For example, inflammatory monocytes contribute to elimination of parasites in *P. chabaudi* infection in mice [2], and in humans, a subset of peripheral monocytes is associated with control of infection in *ex vivo* assays [3]. Additionally, adoptive transfer of a recently discovered progenitor cell that primarily generates monocytes enhances clearance of malaria infection [4]. In contrast, B cells are required for elimination of persistent infection but are dispensable for control of the primary parasitemia [5]–[8]. Similarly, CD8^+^ T cells are not essential for control of blood stage infection [9]. The dispensability of these major effector arms of adaptive immunity highlights the importance of innate mechanisms of anti-parasitic recognition and clearance.

Detection of the offending organism is the critical first step in activating innate immune mechanisms. Many microbes are recognized by innate immune sensors such as Toll-like receptors (TLRs), cytosolic nucleic acid sensors such as RIG-I and MDA5, and nucleotide binding domain-leucine-rich repeat (NBD/LRR) receptors, which can activate downstream production of immunomodulatory cytokines such as the type I interferons alpha and beta (T1IFNs, IFNA, IFNB), tumor necrosis factor (TNF), and interleukin 12 (IL12). In the case of malaria, TLR9 has emerged as a major sensor of infection, although the identity of the ligand remains controversial [4], [10]–[13]. Studies implicating TLR9 in recognition of malaria were conducted using *in vitro*-differentiated plasmacytoid dendritic cells (pDCs), suggesting that pDCs may play a role in *in vivo* recognition of *Plasmodium* infection. This was recently demonstrated to be the case in a report of TLR9-dependent expression of *Ifna* in pDCs during *P. chabuadi* infection of mice [14]. However, it is well known that other innate leukocyte populations, such as conventional dendritic cells (cDCs) and macrophages, also express and signal through TLR9, but the role of these populations in recognition of malaria infection remains largely unexplored.

Although it is clear that detection of malaria infection occurs through TLRs and likely also through other innate immune receptors, the mechanisms through which innate cells contribute to defense against *Plasmodium* parasites are poorly characterized. During viral and bacterial infections, signaling through TLRs and other innate sensing pathways frequently results in the immediate downstream production of cytokines such as T1IFNs. With regard to malaria, *Plasmodium* ligands have been reported to stimulate T1IFN production in *in vitro* systems [10], [13], [15], experimentally infected mice [14], and *Plasmodium*-infected individuals [10], [16]. However, in contrast to IFNG, which has been shown to be an important activator of anti-malarial mechanisms, the role of T1IFNs in protection against malaria infection is not well characterized.

In order to address these gaps in our knowledge, we conducted a systematic investigation of T1IFN production during malaria infection using the *P. chabaudi* model of uncomplicated malaria. Here we present evidence that in addition to pDCs, splenic red pulp macrophages (RPMs) are an important contributor to systemic T1IFN during early malaria infection. Additionally, we have found that T1IFNs regulate gene expression and contribute to control of infection in a manner that is largely redundant with IFNG signaling. However, despite the role of RPMs in T1IFN production, mice lacking RPMs exhibit no deficiencies in their ability to control infection. Our findings demonstrate that T1IFNs play an important immunomodulatory role during *in vivo* malaria infection and provide us with a basic understanding of the molecular and cellular machinery involved in innate immune recognition of malaria parasites. We also demonstrate that RPMs are not essential for control of infection despite their role in early sensing of infection and their key location in contact with circulating parasites.

## Results

### T1IFNs and IFNG Mediate the Early Inflammatory Response to *Plasmodium* Infection

**Figure 1:**
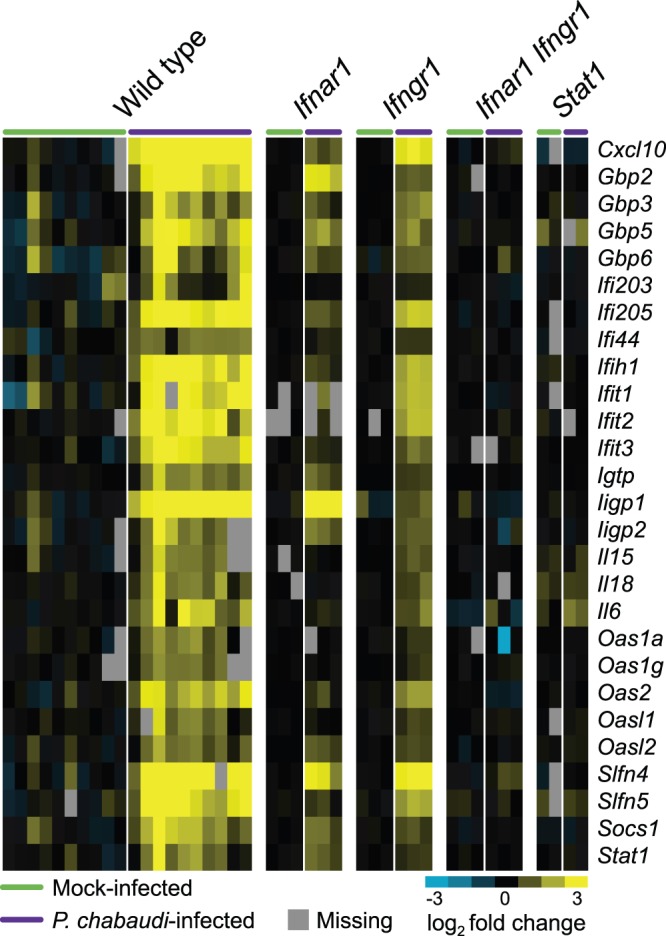
T1IFN and IFNG signaling redundantly regulate early gene expression responses to *P. chabaudi* infection. A representative set of ISG is shown for the gene expression response in whole blood from animals infected for 24 h with *P. chabaudi* in C57BL/6 knockout mice. Each column represents an individual mouse.

We previously reported that genes stimulated as a result of interferon signaling constitute the most extensive gene expression module during the early whole blood response of mice to *P. chabaudi* [17]. In order to identify a highly reproducible signature of early gene expression, we conducted multiple independent gene expression profiling experiments of whole blood of mice infected or mock-infected with *P. chabaudi* at 24 h post-infection. Statistical analysis of the two groups revealed a set of 117 probes (103 unique genes) that were reproducibly increased in relative abundance at 24 h after *P. chabaudi* infection (Table S1). As previously observed, these genes were significantly enriched for known interferon-stimulated genes (ISGs; PANTHER biological process “response to interferon-gamma” *p* = 10^−9^), including classical markers of interferon signaling such as *Cxcl10*, *Il6*, and multiple members of the *Gbp*, *Ifi*, *Ifit*, *Oas,* and *Slfn* gene families (representative genes shown in Fig. 1; complete list available in Table S1).

Members of the two well-characterized classes of interferons, T1IFNs and type II interferon (namely, IFNG), can stimulate cells to induce transcription of ISGs. In order to assess the role of T1IFNs and IFNG in ISG induction in response to *P. chabaudi*, we examined whole blood gene expression signatures in mice deficient in components required for T1IFN and IFNG signaling. In *Ifnar1* ^−*/*−^ mice (deficient in the receptor for T1IFNs), we observed that ISG expression was still induced in response to *P. chabaudi* infection, suggesting that IFNG signaling was a significant mediator of the ISG response. Similarly, *P. chabaudi* infection of *Ifngr1* ^−*/*−^ mice (deficient in IFNG receptor) also resulted in increased ISG transcript abundance compared to mock-infected animals, implying that T1IFN signaling was also contributing to ISG expression during the early response to infection. To determine whether these genes were being induced in a redundant manner, we generated mice doubly deficient in both interferon receptors, and also examined mice deficient in the downstream transcription factor STAT1, which is required for both T1IFN and IFNG signaling. The ISG response in both *Ifnar1* ^−*/*−^ *Ifngr1* ^− */*−^ and *Stat1* ^−*/*−^ animals was completely abolished, demonstrating that both classical interferon signaling pathways act redundantly to induce ISG expression, and that other signaling pathways are not involved.

Although both *Ifnar1* ^−*/*−^ and *Ifngr1* ^−*/*−^ animals were capable of mounting an ISG response, the magnitude of the response in wild type animals appeared to be greater than in either of the immunodeficient strains (41% and 32% average reductions in fold induction by *P. chabaudi* in *Ifnar1* ^−*/*−^ and *Ifngr1* ^−*/*−^ mice, respectively; Fig. S1A). We therefore assessed whether the magnitude of the responses to T1IFNs and IFNG was independent (additive) or redundant (sub-additive). We observed that the sum of the magnitudes of the ISG response in the *Ifnar1* ^−*/*−^ and *Ifngr1* ^−*/*−^ animals was on average greater than the magnitude of the wild type ISG response (slope = 0.7; Fig. S1B), indicating that the T1IFN and IFNG pathways induce the ISG response in a partially redundant manner. Additionally, some redundancy is exhibited even by ISGs that show a degree of preferential induction by T1IFNs or IFNG (Fig. S1C). Although T1IFNs and IFNG are generally thought to mediate different aspects of immune activation, these results demonstrate that at least in the context of early malaria infection, the majority of genes regulated by one type of interferon are also regulated by the other.

**Figure 2:**
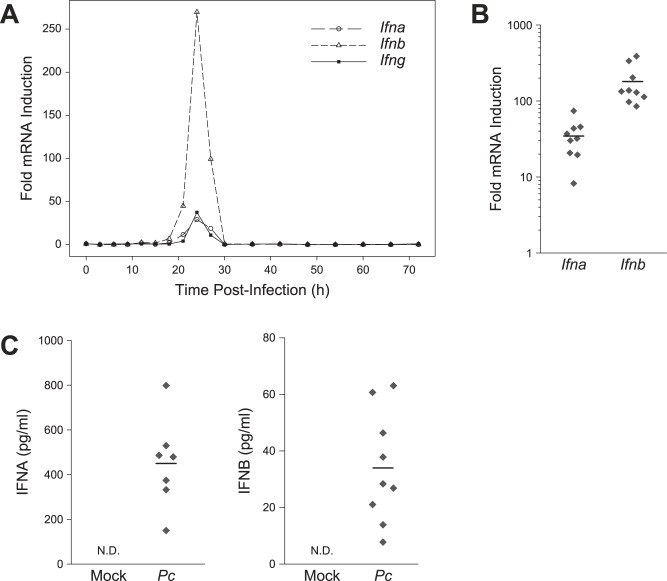
T1IFNs are produced during *P. chabaudi* infection. (A) Kinetics of early *Ifna, Ifnb*, and *Ifng* transcription using whole spleen qRT-PCR. Fold mRNA induction represents the ratio of transcript in infected over mock-infected C57BL/6 mice. (B) Reproducibility of T1IFN transcript induction as detected by whole spleen qRT-PCR. Each point represents an independent experiment with 4–6 animals, with horizontal bars displaying the geometric mean. (C) Plasma IFNA and IFNB at 24 h post-infection. Data are combined from two independent experiments with each point representing one animal. N.D. = not detected (*n* = 8).

In order to directly measure T1IFN production, we performed quantitative reverse transcription PCR (qRT-PCR) to estimate relative transcript abundance for *Ifna* and *Ifnb* in the spleens of mice infected with *P. chabaudi*. Examination of splenic transcripts every 3 h for the first 30 h post-infection revealed that both *Ifna* and *Ifnb* transcripts, as well as *Ifng*, exhibited a peak of increased abundance centered around 24 h (Fig. 2A). Upon return to baseline levels, splenic T1IFN transcripts were not induced again within the first three days of infection (measured in 6 h intervals after 30 h). Detection of elevated *Ifna* and *Ifnb* in spleens of infected animals at 24 h post-infection was highly reproducible across independent experiments (Fig. 2B), and IFNA and IFNB were reproducibly detected in the plasma of infected animals (Fig. 2C). Together, these findings provide evidence that a burst of T1IFNs is produced during the early response to *P. chabaudi* infection and contributes to induction of ISG expression.

### T1IFNs and IFNG Redundantly Promote Control of Parasitemia

**Figure 3:**
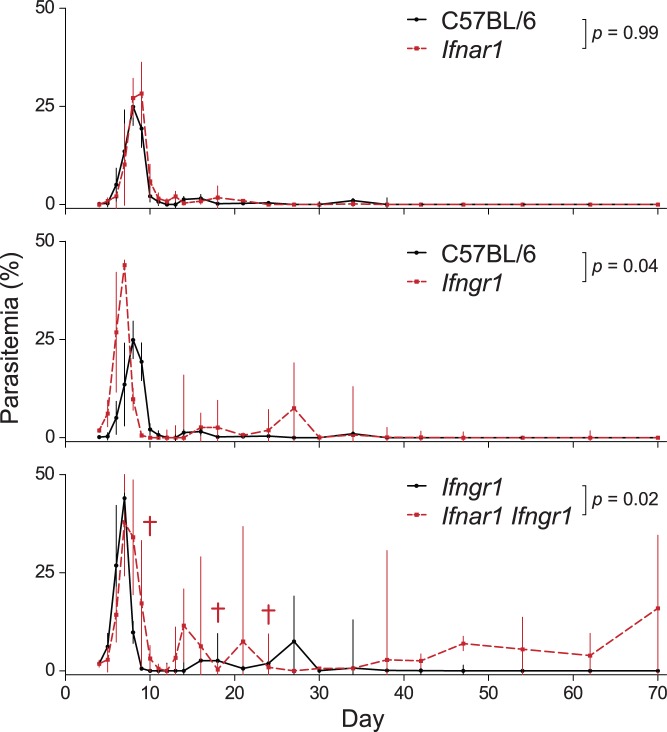
T1IFNs contribute to control of *P. chabaudi* infection. Infected mice were monitored for parasitemia by thin blood smear and survival. Wild type C57BL/6 and congenic knockout mice were infected and monitored for percent parasitemia (*n* = 5 per strain), which are represented as geometric means with standard deviations and Mann-Whitney *p*-value. A representative experiment of two is shown. Crosses indicate deaths due to parasitemia.

Our results show early production of T1IFNs during *P. chabaudi* infection, but the contribution of T1IFNs to the control of malaria parasite replication is not well characterized. The normal course of *P. chabaudi* infection in C57BL/6 mice develops as an exponentially increasing load of parasites in the blood that typically peaks at 7–10 days post-infection followed by control and resolution of the primary parasitemia over the next 2–4 days (Fig. 3). A recent study reported a slight increase in the magnitude of peak *P. chabaudi* parasitemia in *Ifnar1* ^−*/*−^ mice, but resolution occurred with kinetics identical to wild type (129Sv) animals [14]. In contrast, we observed no significant differences in the magnitude or times to manifestation of any of the ascending, descending, or clearance phases of parasitemia in *Ifnar1* ^−*/*−^ animals as compared to infection of C57BL/6 mice (Fig. 3). The discrepancy between our findings and those of Voisine *et al.* could be a result of the different backgrounds used, since 129Sv mice produce higher levels of T1IFNs (Fig. S3 and [18]).

Although our results would appear to suggest that T1IFNs do not contribute to control of malaria infection, we considered the possibility that the redundancy between T1IFNs and IFNG in the regulation of ISG expression could confer redundancy in control of infection. We therefore examined the course of parasitemia in *Ifngr1* ^−*/*−^ animals as compared to *Ifnar1* ^−*/*−^ *Ifngr1* ^−*/*−^ animals in order to assess the function of T1IFNs in the absence of IFNG signaling. We observed that *Ifngr1* ^−*/*−^ animals exhibited defects in their ability to resolve parasitemia as compared to wild type animals; although most animals controlled the primary and secondary peaks, peak parasitemias were higher in *Ifngr1* ^−*/*−^ animals, and a tertiary peak of parasitemia occurred in most animals (Fig. 3). Despite the increased severity of infection in *Ifngr1* ^−*/*−^ mice, parasites were controlled in all mice by 40 days post-infection. In contrast, *Ifnar1* ^−*/*−^ *Ifngr1* ^−*/*−^ animals exhibited mortality, multiple late peaks of high parasitemia, and an inability to completely clear parasites from the bloodstream within the duration of the 70 day study, indicating that T1IFNs and IFNG signaling exhibit redundancy in the regulation of anti-parasitic mechanisms that are essential to the control of malaria infection.

### Plasmacytoid Dendritic Cells and Red Pulp Macrophages Produce T1IFNs in Response to *P. chabaudi*

Previous *in vitro* studies have provided conflicting measurements of production of IFNA by pDCs after stimulation with malaria ligands [10]–[13]. Another study recently reported *Ifna* expression in pDCs during *P. chabaudi* infection [14], but this observation was made at a time after the peak of C57BL/6 T1IFN production and did not assess other potential cellular sources. We therefore took an unbiased approach to identify the cellular origins of T1IFN production in response to physiologically relevant stimuli during *in* *vivo* infection with *P. chabaudi*.

**Figure 4:**
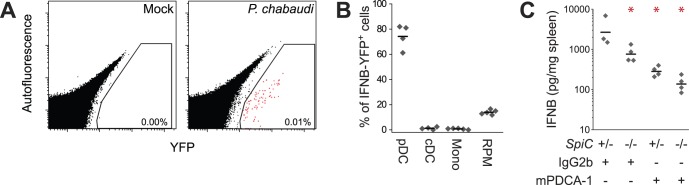
*P. chabaudi* infection induces IFNB production in pDCs and RPMs. (A) No splenocytes are YFP^+^ in mock-infected samples, but some splenocytes become YFP^+^24 h after *P. chabaudi* infection of C57BL/6 *Ifnb* reporter mice. 2.5×10^6^ events are depicted in each dot plot. (B) pDCs and RPMs constitute over 90% of YFP^+^ events in C57BL/6 mice. (C) Both pDCs and RPMs contribute to splenic IFNB production in 129Sv mice. pDCs were depleted with a single 500 µg dose of anti-mPDCA1 antibody 18 h before infection with *P. chabaudi*. Grey dots represent individual mice, with horizontal bars representing the mean (B) or geometric mean (C).

In order to achieve single-cell resolution of T1IFN expression, transgenic *Ifnb-Yfp* reporter mice [19] were mock- or *P. chabaudi*-infected and analyzed for splenic *Ifnb* expression by flow cytometry. Animals infected with *P. chabaudi* contained a small but highly reproducible population of YFP^+^ cells, whereas no YFP^+^ events were detected in any of the spleens of mock-infected animals (Fig. 4A). Lineage marker analysis of the YFP^+^ populations demonstrated that approximately 75% of the YFP^+^ events were CD11c^int^ Siglec-H^+^, consistent with markers of pDCs (Fig. 4B). In contrast, conventional dendritic cells (cDCs; CD11c^hi^ Siglec H^−^) and CD11b^hi^ F4/80^int-hi^ SSC^lo^ monocytes (Mono) constituted none of the YFP^+^ events. Interestingly, a small but reproducible fraction (∼15%) of the total YFP^+^ events was F4/80^hi^ CD11b^lo/−^, consistent with markers of splenic RPMs. Similar frequencies of YFP^+^ and lineage markers were observed using *Ifna6-Gfp* reporter mice (Fig. S2) [20]. Notably, the pDCs and RPMs together account for nearly all the YFP^+^ and GFP^+^ cells, indicating that, together, they are the major populations responsible for splenic T1IFN induction during *P. chabaudi* infection.

Because T1IFN can be produced at low levels by other cell types, we assessed whether pDCs and RPMs measurably contribute to systemic T1IFN levels. In order to examine the role of RPMs in T1IFN production, we employed *SpiC* ^−*/*−^ mice [21], which lack a transcription factor required for development of RPMs but not other myeloid populations (Fig. S3A). pDCs were depleted 18 h pre-infection with *P. chabaudi* using the anti-mPDCA-1 antibody, which reproducibly depleted 85% of splenic pDCs with no impact on RPM frequency (Fig. S3B). After 24 h infection with *P. chabaudi*, *SpiC* ^−*/*−^ mice produced roughly half the splenic IFNB of *SpiC^+/^* ^−^mice (Fig. 4C), with similar results also observed in plasma (Fig. S3C–D). pDCs were also required for T1IFN production, with depletion resulting in over 80% reduction of splenic and plasma IFNB levels in *SpiC^+/^* ^−^ mice (Fig. 4C and S4D) and C57BL/6 mice (Fig. S4E). The absence of both populations resulted in over 90% reduction of splenic IFNB (Fig. 4C), with the residual levels likely reflecting incompletely depleted pDCs. Together with the reporter data, these results demonstrate that pDCs and RPMs are the primary sources of T1IFN during experimental malaria infection.

### Red Pulp Macrophages are the Primary Source of Splenic T1IFN Transcripts during *P. chabaudi* Infection

**Figure 5:**
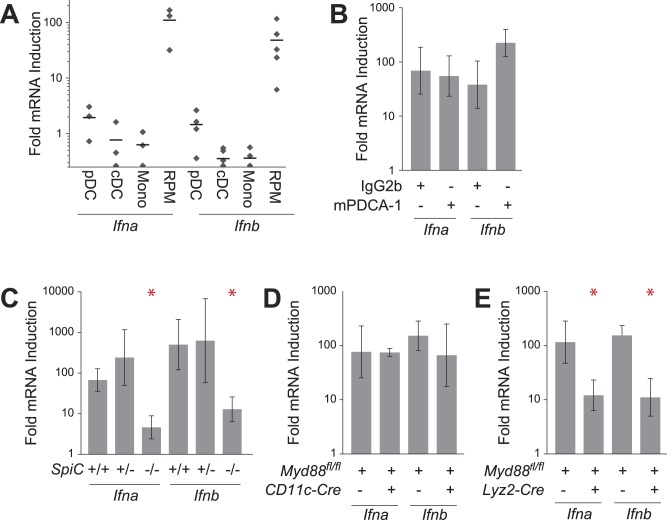
Cellular requirements for splenic T1IFN transcriptional induction. (A) RPMs, but not other macrophage or dendritic cell subsets, induce *Ifna* and *Ifnb* at 24 h post-infection with *P. chabaudi* as detected by qRT-PCR in C57BL/6 mice. Fold mRNA induction represents fold induction of transcript in infected versus mock-infected normalized to beta-actin. Grey dots represent independent experiments conducted on different days; black bars denote the geometric means of the fold inductions. (B) pDCs are not required for splenic *Ifna* or *Ifnb* transcriptional induction in response to *P. chabaudi* in C57BL/6 mice. (C) Genetic deletion of RPMs in 129Sv mice results in diminished T1IFN transcriptional induction. (D) Genetic deletion of *Myd88* from dendritic cells does not impact transcriptional induction of T1IFNs in spleens of C57BL/6 mice. (E) Genetic deletion of *Myd88* from macrophages/neutrophils decreases transcriptional induction of T1IFNs by an order of magnitude in C57Bl/6 mice. Grey bars represent geometric means with 95% confidence intervals. Asterisks represent *p*<0.05 in a Student’s *t*-test against control samples.

In order to corroborate our observations with the *Ifnb* reporter mice, we isolated the same splenic leukocyte subsets by FACS and assessed T1IFN transcriptional induction by qRT-PCR. Consistent with our observations in *Ifnb-Yfp* reporter mice, RPMs strongly induced both *Ifna* and *Ifnb* transcript post-infection with *P. chabaudi* (Fig. 5A). Similarly, microarray analysis of isolated RPMs from mock- and *P. chabaudi-*infected mice demonstrated induction of multiple members of the *Ifna* family along with a variety of other cytokines and chemokines, including *Tnf, Il1b, Il6, Il10, Cxcl1,* and *Cxcl2* (Fig. S4A), and RPM-deficient mice exhibited decreased plasma levels of TNFA and IL12p70 (Fig. S4B). These results demonstrate that RPMs activate a diverse repertoire of immunomodulatory products, including T1IFNs, during the early response to *P. chabaudi* infection.

In contrast to RPMs, splenic pDCs surprisingly did not demonstrate any significant induction of T1IFN transcript as measured by qRT-PCR. This was not a result of elevated baseline T1IFN transcript levels as observed in other studies (Fig. S5) [22]. We also did not detect elevated T1IFN in pDCs at earlier time points, which is consistent with our observation that splenic T1IFN transcript abundance peaks at 24 h (Fig. 2A). Because YFP has a very long half life, we speculate that YFP^+^ splenic pDCs have become activated at a slightly earlier time and in a different compartment before migrating to the spleen, consistent with their role as sentinel cells that can migrate to sites of inflammation [23]. The results also suggest that RPMs are the population primarily responsible for induction of T1IFN transcription in the spleen, although both RPMs and pDCs contribute to production of circulating plasma T1IFN.

To further assess the role of pDCs in T1IFN induction in *P. chabaudi*-infected mice, we depleted pDCs as above and measured splenic T1IFN transcript at 24 h post-infection. Animals depleted of pDCs were intact in their ability to induce splenic transcription of either *Ifna* or *Ifnb* as compared with IgG2b-treated control animals (Fig. 5B). These results indicate that pDCs do not contribute to the splenic T1IFN transcript pool at 24 h post-infection, despite the fact that they are responsible for the majority of circulating T1IFN protein.

In a complementary approach, we examined the role of RPMs in transcriptional induction of T1IFNs using *SpiC* ^−*/*−^ mice. Wild type 129Sv and *SpiC^+/^* ^−^ mice both strongly induced splenic T1IFN transcription in response to *P. chabaudi* infection (Fig. 5C). In contrast, T1IFN transcriptional induction was reduced by over 90% in *SpiC* ^−*/*−^ animals, which we note is similar to the extent of RPM depletion in *SpiC* ^−*/*−^ mice [21]. Together, these results confirm that transcriptional induction of T1IFNs in the spleens of *P. chabaudi*-infected mice primarily occurs in RPMs, but that systemic T1IFN is produced by both RPM and pDCs.

In a final approach to characterizing the cellular origins of splenic T1IFN transcriptional induction, we employed mice homozygous for a floxed allele of *Myd88* (*Myd88^fl/fl^*), which encodes an adaptor molecule required for TLR signaling. *Myd88^f l/fl^* mice that are hemizygous for the *CD11c-Cre* or *Lyz2-Cre* transgene efficiently delete *Myd88* from the genomes of dendritic cells and macrophages, respectively [22]. Consistent with the model that RPMs are responsible for transcriptional induction of T1IFNs in spleens, we observed normal levels of splenic *Ifna* and *Ifnb* transcription in *CD11c-Cre* animals, whereas *Lyz2-Cre* animals exhibited a nearly 90% reduction in T1IFN transcription (Fig. 5D–E). These data indicate that *Myd88* is required in macrophages, but not in dendritic cells, for transcriptional induction of splenic T1IFNs. Taken together, our data lead us to conclude that RPMs, and not pDCs, are the primary source of splenic T1IFN transcripts at 24 h after *P. chabaudi* infection, despite the fact that both populations contribute IFNB to the circulating plasma pool.

### TLR9 Signaling is Required for Full Induction of T1IFNs

**Figure 6:**
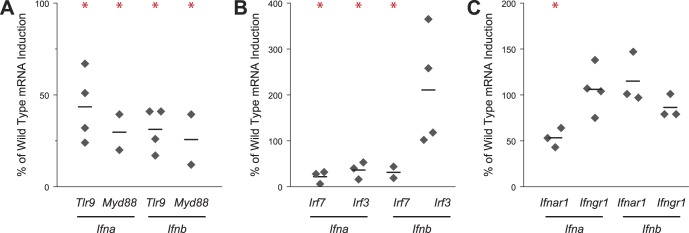
Molecular requirements for splenic T1IFN transcriptional induction. (A) *Tlr9* and *Myd88* are required for full transcriptional induction of T1IFNs in spleens of *P. chabaudi*-infected C57Bl/6 mice. Grey dots represent the means of independent experiments using 4–6 total mice, with T1IFN fold mRNA induction in knockout mice represented as a percentage of induction in wild type animals. Black bars represent means; asterisks represent *p*<0.05 as compared to wild type induction in a two-tailed Student’s *t*-test assuming unequal variances. (B) *Irf7* is required for full T1IFN induction, and *Irf3* is required for full *Ifna* but not *Ifnb* induction. (C) *Ifnar1* is required for full *Ifna* induction but dispensable for *Ifnb* induction, whereas *Ifngr1* is dispensable for all T1IFN induction.

Activation of TLR9 by A-type CpG DNA leads to induction of IFNA in pDCs through a MYD88- and IRF7-dependent mechanism [24], and previous work similarly found TLR9-dependence of IFNA production in pDCs during *in vitro* infection with malaria parasites [14]. To characterize the molecular mechanisms by which RPMs respond to *Plasmodium* infection, we took advantage of the fact that splenic T1IFN transcript is almost exclusively derived from RPMs to examine the role of several signaling molecules. We measured the induction of splenic T1IFN transcript by qRT-PCR in wild type and knockout mice infected with *P. chabaudi* for 24 h. In *Tlr9* ^−*/*−^ mice, we consistently observed a two- to four-fold decrease in production of *Ifna* and *Ifnb* transcript compared to wild type mice (Fig. 6A). Consistent with an important role for TLR9 signaling in T1IFN production, *Ifna* and *Ifnb* transcripts failed to be induced to wild type levels in *Myd88* ^−*/*−^ animals, similar to our results in *Lyz2-Cre Myd88^fl/fl^* mice (Fig. 6A and 5D). Mice harboring the *Ifnb-Yfp* reporter gene in addition to a deficiency in either *Myd88* or *Unc93b1* (required for TLR3, TLR7, and TLR9 signaling [25]) also failed to induce the *Ifnb-Yfp* reporter in response to *P. chabaudi* infection (Fig. S6). These results indicate that TLR9 and MYD88 contribute to the *P. chabaudi*-induced transcription of T1IFNs not only in pDCs [14], but also in RPMs (Fig. S6). We note that T1IFN induction in *Myd88* ^−*/*−^ animals as measured by qRT-PCR is not completely abrogated, suggesting the existence of a MYD88-independent pathway for T1IFN induction.

In pDCs, IFNA production as a result of TLR9 activation by CpG is strongly dependent on the transcription factor IRF7 [24]. We observed that RPM from *Irf7^−/−^* mice also exhibit decreased splenic T1IFN transcription in response to *P. chabaudi* (Fig. 6B). We also assessed the role of the transcription factor IRF3 in T1IFN production since TLR signaling in macrophages can generate an early wave of T1IFN that is dependent on IRF3-mediated initiation of an IFNAR1- and IRF7-dependent autocrine amplification loop [26]. RPM from *Irf3^−/−^* mice demonstrated no defect in *Ifnb* induction in response to *P. chabaudi*. In contrast, *Irf3^−/−^* mice exhibited a diminished (two- to three-fold) capacity for *Ifna* expression compared with wild type animals. In addition to implicating IRF3 activation in *Ifna* production, these results indicate that the regulatory mechanisms of *Ifna* and *Ifnb* induction in response to *P. chabaudi* have distinct requirements, as also observed in macrophages responding to West Nile Virus [27].

Finally, we assessed the possibilities that a T1IFN amplification loop or crosstalk with IFNG could influence T1IFN induction by *P. chabaudi*. We observed that *Ifnar1* is required for wild type levels of expression of *Ifna* but not *Ifnb*, suggesting that *Ifna* (but not *Ifnb*) may be amplified through an amplification loop (Fig. 6C). In contrast, *Ifngr1* ^−*/*−^ animals exhibit no defects in T1IFN induction, ruling out the possibility of crosstalk from IFNG signaling. Taken together, the results suggest a model in which IFNA expression in RPMs is dependent on IRF3 and on an amplification loop requiring IFNAR1, whereas IFNB induction is independent of IRF3 and the T1IFN amplification loop.

### Red Pulp Macrophages are Dispensable for Control of Parasitemia

**Figure 7:**
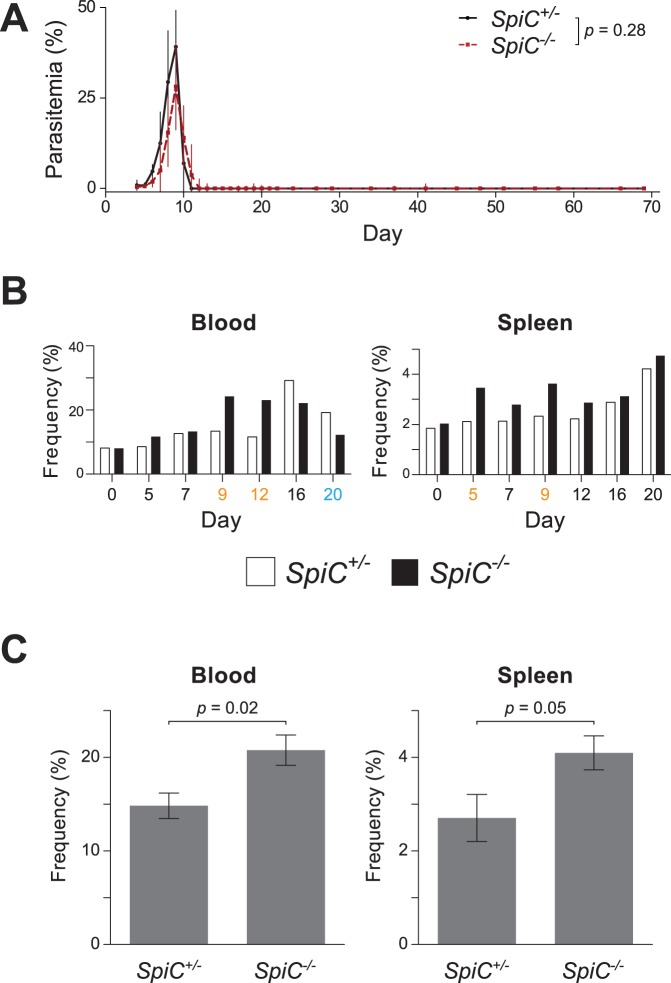
Mice lacking RPMs exhibit wild type infection kinetics. (A) Parasitemia courses in 129Sv *SpiC^+/−^* (*n* = 4) and *SpiC^−/−^* (*n* = 5) mice are represented as geometric means with standard deviations and Mann-Whitney *p*-value. (B) Ly6c^lo^ monocyte (CD11b^+^ F4/80^+^ Ly6g^−^ SSC^lo^ Ly6c^lo^) frequencies in blood and spleen of 129Sv *SpiC^+/−^* (white bars) and *SpiC^−/−^* mice (black bars) during the course of infection. Days depicted in blue and orange represent a 1.5-fold decrease or increase, respectively, in Ly6c^lo^ monocyte frequencies in blood and spleen of mice infected with *P. chabaudi*. (C) Ly6c^lo^ monocyte frequencies on day 12 post-infection. Means are presented with standard errors; *p*-values represent a two-tailed *t*-test assuming unequal variances. Data represent three independent experiments (*n* = 6–7 mice per group total).

Previous work using 120G8-derived antibodies to deplete pDCs has demonstrated that these cells are dispensable for control of *P. chabaudi* infection [14], and we have made similar observations using the anti-mPDCA-1 pDC-depleting antibody (Fig. S7A). In contrast, it is generally believed that RPM play an important role in parasite control given their association with circulating malaria parasites [28], their ability to phagocytose “pitted” parasites and parasitized erythrocytes [29], their expansion during infection [30], [31], the exacerbation of experimental malaria infection upon phagocyte depletion [32], [33], and the contribution of monocyte-derived splenic leukocytes to parasite elimination [2], [4]. Together with our own observation that RPMs are early sentinels of infection, the above evidence led us to hypothesize that mice lacking RPMs would exhibit increased susceptibility to infection. Surprisingly, the course of rising and falling parasitemia in *SpiC^− /−^* mice occurred with kinetics essentially identical to the course in *SpiC^+/−^* animals (Fig. 7A) and wild type C57BL/6 animals (Fig. 3A and 3B). In order to explore the possibility that another myeloid population was compensating for the lack of RPMs, we enumerated major myeloid populations in the blood and spleen of *SpiC^+/−^* and *SpiC^−/−^* mice over the course of *P. chabaudi* infection. No consistent differences were observed in the frequencies of neutrophils (CD11b^hi^ Ly6g^+^), cDCs, pDCs, or splenic marginal zone macrophages (CD11b^−^ F4/80^−^ MARCO^+^) (Fig. S7B-C). In contrast, Ly6c^lo^ monocyte frequencies were increased in the blood of *SpiC^−/−^* mice during resolution of peak parasitemia (days 9 and 12), and were significantly higher in spleens of *SpiC^−/−^* mice throughout infection (Fig. 7B *; p* = 0.02, Wilcoxon matched pairs signed-rank test). Detailed examination of monocyte frequencies on day 12 post-infection confirmed that Ly6c^lo^ monocytes were significantly elevated in both blood and spleens of *SpiC^−/−^* mice (Fig. 7C). We therefore conclude that although RPM contribute to early immune infection recognition and activation and are well positioned to interact with parasites, they are ultimately dispensable for control of infection, possibly as a result of compensation by Ly6c^lo^ monocytes.

## Discussion

We previously found that *P. chabaudi* infection of mice induced robust expression of an interferon-induced gene signature as the earliest detectable expression response in blood [17]. Here, we demonstrate that this ISG response is the combined result of T1IFNs and IFNG, acting in a largely redundant fashion. Although the prominent involvement of IFNG in responses to malaria infection is well established, much less is understood about production of T1IFNs. Studies have shown that malaria extracts can induce IFNA from human pDCs *in vitro* [10], [13], and have documented IFNA induction in *P. chabaudi*- [14] and *P. berghei*-infected mice [34]. Using a variety of approaches, we have demonstrated that T1IFNs are indeed produced during *in vivo* infection with *P. chabaudi*, and that both pDCs and RPMs are the key cellular sources that contribute to the systemic T1IFN pool.

Although the protective role of T1IFNs in viral infections is well established, in some bacterial infections and autoimmune disorders, T1IFNs appear to exacerbate disease [35]. Similar to viral infections, our functional studies indicate that T1IFNs act redundantly with IFNG to activate mechanisms that protect against malaria disease. Together, our findings reveal redundancies at several different levels: first, at the level of multiple molecular sensing pathways in RPMs feeding into T1IFN production; second, at the level of multiple leukocyte populations generating systemically available T1IFNs; and finally, at the level of T1IFNs conferring protection that is redundant with IFNG. We suggest that this tiered redundancy is widespread in immunological systems but has been overlooked due to absent or mild phenotypes in organism-level assays.

T1IFNs frequently originate from pDCs, which are also known as “interferon producing cells” due to their ability to produce more T1IFNs than any other cell type in human blood [36]. Our observation that pDCs produce IFNA and IFNB during malaria infection is in line with the general function of pDCs and similar findings from Voisine *et al.* [14]. However, we have demonstrated that RPMs also contribute significantly to total T1IFN production during the response to *P. chabaudi*, indicating that these macrophages play a role in early immune activation during malaria infection. We estimate that roughly 3000 pDCs and 1000 RPMs per spleen produce high levels of T1IFN, and the comparable fluorescence levels of these populations in *Ifnb-Yfp* reporter animals suggest that pDC and RPM are capable of transcribing similar levels of *Ifnb*. Whether or not this corresponds to similar levels of IFNB production on a per-cell basis remains to be determined; regardless, our findings contribute to the increasing body of literature indicating that macrophages and other non-pDC populations are significant sources of T1IFNs *in vivo* [27], [37]–[41].

It is likely that the localization of the infections at the tissue, cellular, and sub-cellular levels defines in part which leukocytes respond and in what manner. This is likely to be the case for T1IFN production by RPMs in malaria infection: ultrastructural studies have demonstrated that RPMs are capable of phagocytosis of both whole infected erythrocytes and parasites that have been “pitted” from infected erythrocytes in the spleen [29], and trafficking studies using stained infected erythrocytes have demonstrated localization to the splenic red pulp [28]. Although these studies only examined splenic organization during the time of peak parasitemia, it was reasonable to expect that RPMs would also function as early detectors of malaria parasites due to their inherent role in filtering parasites from the blood. We have demonstrated that this is indeed the case, despite the low parasite load during early sub-patent infection, and that RPMs respond by producing T1IFNs and a host of additional chemokines and cytokines. To the best of our knowledge, this is the first demonstration of production of an immunomodulatory cytokine by RPMs during early malaria infection.

We have found that TLR9-MYD88-IRF7 signaling is required for full T1IFN expression in RPMs, similar to the role of this pathway in pDC [14]. This is at odds with the fact that no *in vitro* studies of TLR9 activation have reported IFNA production in mouse pDCs or macrophages, but it is possible that malaria ligands may be less potent than synthetic ligands and therefore require additional activating signals from other leukocyte populations present *in vivo*. Obvious candidates for such signals include cytokines that signal through the MAP kinase and NF-kappa B pathways, which participate in *Ifnb* induction through the heterodimeric transcription factors ATF-2/c-Jun and p50/RelA [42]. Consistent with this possibility, inhibition of NF-kappa B signaling in mice infected with West Nile Virus decreases IFNB production [27]. Further studies will be required to understand the relative contributions of these pathways *in vitro* and *in vivo*, and also to identify the pathway(s) responsible for residual levels of T1IFN production in the absences of TLR9 and MYD88.

T1IFNs can augment their own expression through a feed-forward signaling loop, but for *P. chabaudi*, only *Ifna,* not *Ifnb,* induction appears to rely on IFNAR1-dependent amplification. This result is similar to observations from *Listeria* infection, in which IFNB generation is essentially unaffected by the absence of IFNAR1 whereas IFNA production is severely diminished [40]. Similarly, expression of *Ifna* by cDCs during West Nile Virus infection was diminished in mice lacking IFNAR1, whereas *Ifnb* expression was not [27]. Thus, our data extend the paradigm of IFNB being induced prior to amplification loop-dependent production of IFNA, as demonstrated in viral and bacterial systems, to infection with a protozoan parasite. With regard to *Ifna* induction by *P. chabaudi*, we observed that *Ifnar1* ^−*/*−^and *Irf3* ^−*/*−^ mice exhibit similar levels of reduction, consistent with observations from other systems that these molecules are both required for T1IFN amplification [26].

Although RPMs produce T1IFNs and other cytokines during early infection, mice lacking RPMs clear parasites with kinetics identical to control animals. This result was surprising given the general belief that RPMs contribute to control of parasitemia through phagocytic mechanisms [28], [29], [33], [43]. Furthermore, we observed that RPMs act as early sentinels of infection and produce cytokines that ultimately contribute to elimination of infection. Given our observation that pDCs also produce T1IFNs, it is possible that all of the important functions of RPMs are redundant with other leukocyte subsets. For example, splenic monocytes are capable of phagocytosis of *P. chabaudi* [2], and this population undergoes expansion near the time of peak parasitemia in both *SpiC^+/^* ^−^ and *SpiC* ^−*/*−^ mice (Fig. S7C). Our data indicate that the Ly6c^lo^ monocytes are also significantly increased in frequency in RPM-deficient mice, suggesting the possibility that this subset could be providing redundancy with RPMs. Although the exact mechanism requires further investigation, our data indicate that the important role of the spleen in clearance of malaria infection is due to functions that are not specific to RPMs.

In summary, our results demonstrate that T1IFNs play a redundant but important protective role during experimental malaria infection. These T1IFNs are derived from both pDCs and RPMs, which are thus identified as the major populations responsible for early innate recognition of malaria infection. Future work will reveal how these innate populations and T1IFNs promote the development of an integrated immune response that can ultimately resolve malaria infection.

## Materials and Methods

### Mice

C57BL/6 9–14 week old female mice (Jackson Laboratories or National Cancer Institute) were maintained on a 12 h light cycle (on from 0600 to 1800 h). All mice used in this study (*Ifnar1* ^−*/*−^, *Ifngr1* ^−*/*−^, *Ifnar1* ^−*/*−^ *Ifngr1* ^−*/*−^, *Stat1* ^−*/*−^, *Ifnb-Yfp^+/+^, Ifna6-gfp^+/^* ^−^, *Tlr9* ^−*/*−^, *Myd88* ^−*/*−^, *Irf7* ^−*/*−^) were >95% C57BL/6 by microsatellite genotyping at 94 loci (UCSF genomics core) with the exceptions of *Irf3* ^−*/*−^ (80% C57BL/6) and *SpiC^−/−^* mice (129Sv). This study was conducted in strict accordance with the guidelines of the Office of Laboratory Animal Welfare and with the approval of the UCSF Institutional Animal Care and Use Committee.

### Parasites

*P. chabaudi* AS (MRA-429) was maintained in C57BL/6 mice. Blood was harvested by cardiac puncture from an infected mouse just prior to peak parasitemia and 10^6^ infected erythrocytes were introduced by intraperitoneal injection. All infections were initiated at 1400 h. Blood was harvested by cardiac puncture, and spleens were harvested for analysis at specified times.

### RNA

Samples for RNA preparation were immersed in RNAlater (Ambion) upon harvest and stored at -80°C. RNA from blood was isolated by using the Mouse Ribopure-Blood kit (Ambion) and amplified in a single round using the Amino Allyl MessageAmp II aRNA Amplification Kit (Ambion). RNA from spleens was isolated using Trizol as per the manufacturer’s protocol, followed by two rounds of treatment with Turbo DNase (Ambion). RNA from FACS-sorted leukocyte subsets was isolated and treated with DNase using the RNAqueous Micro Kit (Ambion).

### Microarrays

All microarray methods used in this study were as previously described [17]. Further details are provided as supplementary material. Data are available through the Gene Expression Omnibus (GSE23565).

### qRT-PCR

For splenic RNA analysis by qRT-PCR, 3 µg of RNA was reverse transcribed, diluted, and amplified with Quantitect SYBR Green (Qiagen) on an Opticon thermal cycler (MJ Research). Sorted leukocyte RNA was processed similarly except the entire RNA sample was used in the RT. “Universal” primers were designed to target multiple *Ifna* variants (GTGAGGAAATACTTCCACAG, GGCTCTCCAGACTTCTGCTC). Primers for *Act* (GGCTGTATTCCCCTCCATCG, CCAGTTGGTAACAATGCCATGT) and *Ifnb* (CAGCTCCAAGAAAGGACGAAC, GGCAGTGTAACTCTTCTGCAT) were from PrimerBank [44]. T1IFN transcript levels were normalized to beta-actin levels and fold-inductions calculated using the Pfaffl method.

### ELISA

Assays for IFNA and IFNB were performed as per the manufacturer’s instructions (Pestka Biomedical Laboratories) on K_2_EDTA plasma or spleens homogenized in PBS with a protease inhibitor cocktail (Roche) using a TissueLyzer II (Qiagen).

### Flow Cytometry

Spleens were mechanically homogenized in FACS buffer. Erythrocytes were lysed in 1x RBC lysis solution. Fc receptors on the leukocytes were blocked with anti-CD16/CD32 antibody (2.4G2; UCSF hybridoma core), stained with specific antibodies, and analyzed/sorted on an LSR II or FACSAria II. Antibodies used for leukocyte subset identification included those targeting Siglec H (eBio440c), Ly6c (HK1.4), CD11c (N418), and rat IgG1 staining control (eBioscience); F4/80 (BM8), CD11b (M1/70), Ly6g (1A8), and rat IgG2a staining control (2A3) (UCSF hybridoma core); and MARCO (ED31; Thermo Fisher).

## Supporting Information

Figure S1 **Redundancy and specificty in interferon signaling.** (A) The distribution of percent reduction in fold-induction for individual ISG in IFN receptor knockout mice. (B) The sum of the average magnitudes of T1IFN and IFNG gene induction amount to more than the whole observed in wild type mice, indicating redundancy in gene expression. Each point represents a different probe, and lines represent the linear regression and 95% confidence interval. (C) A subset of ISG exhibit preferential induction by either T1IFN or IFNG. The log_2_ fold induction of the 117 early response genes is plotted for *Ifnar1^−/−^* and *Ifngr1^−/−^* mice to identify preferentially induced genes. Residuals from identity (x = y) were calculated, and an arbitrary cutoff of 1.4 was chosen to highlight the most distant genes (*i.e.* the most preferentially induced genes). Green points represent genes preferentially induced by IFNG, and red points denote genes preferentially induced by T1IFN.
(PDF)

Figure S2 **Induction of** ***Ifna6-Gfp*** **expression in splenic leukocytes by** ***P. chabaudi*** **.** GFP^+^ events were analyzed for lineage markers 24 h after infection as in Fig. 4.
(PDF)

Figure S3 **Both pDCs and RPMs are required for full T1IFN production during** ***P. chabaudi*** **infection.** (A) Live singlet cells were subjected to lineage marker analysis for myeloid populations, demonstrating that *SpiC^−/−^* mice exhibit reduced RPM frequency compared to *SpiC^+/−^* animals, but otherwise have intact splenic macrophage and dendritic cell populations. (B) Treatment of C57BL/6 mice with mPDCA-1 antibody depletes splenic pDC populations but does not affect red pulp macrophages. Data represents frequencies measured after 18 h depletion plus 24 h infection with P. chabaudi. (C) Plasma IFNB levels are diminished in 129Sv *SpiC^−/−^* compared to 129Sv *SpiC^+/−^* mice. (D) Deficiencies in RPM and pDCs diminish the plasma IFNB response to *P. chabaudi* in 129Sv *SpiC^−/−^* mice. (E) Depletion of pDCs in C57BL/6 mice decreases the plasma IFNB response to *P. chabaudi*. Asterisks represent *p*<0.05 in a two-tailed *t*-test assuming unequal variances compared with intact controls.
(PDF)

Figure S4 **RPMs induce expression of** ***Ifna*** **and other cytokines and chemokines in response to** ***P. chabaudi*** **infection.** (A) RNA was harvested from FACS-isolated RPMs from mock- or *P. chabaudi*-infected C57BL/6 animals, amplified, and hybridized to microarrays. A representative set of cytokines and chemokines induced upon infection are shown with fold change in transcript abundance. (B) Plasma cytokines of 129Sv *SpiC^+/−^* and *SpiC^−/−^* mice infected for 24 h with *P. chabaudi* were measured using Milliplex analysis (Millipore) on a MagPix instrument (Luminex). Differences between *SpiC^+/−^* and *SpiC^−/−^* mice are significant by a two-tailed *t*-test assuming unequal variances (α = 0.05; red asterisks).
(PDF)

Figure S5 **Basal C(t) values for leukocyte subsets.** FACS-sorted populations from mock-infected animals were subjected to qRT-PCR for T1IFN transcripts. The data were aggregated from 4 independent experiments, with means and 95% confidence intervals represented. No significant differences were observed for any populations.
(PDF)

Figure S6 **MYD88 is required for** ***Ifnb-Yfp*** **induction.** Mice were inoculated with 10^6^ infected erythrocytes or mock-infected with uninfected erythrocytes and spleens were harvested and processed for flow cytometry 24 h later.
(PDF)

Figure S7 **pDC and RPM are both dispensable for the control of** ***P. chabaudi*** **parasitemia.** (A) C57BL/6 mice were intraperitoneally infected with 10^6^ parasites. On day 4 post-infection, 500 µg of anti-mPDCA-1 antibody (Miltenyi Biotec) or IgG2b isotype control antibody (LTF2, UCSF hybridoma core) was administered intraperitoneally. Parasitemias are presented as geometric means with standard deviations and Mann-Whitney *p*-value. (B) Gating strategy for identification of myeloid populations in blood and spleen. Live singlet cells (not shown) were subjected to lineage marker analysis. MZM = marginal zone macrophages. (C) Myeloid population frequencies in blood and spleens of 129Sv *SpiC^+/−^* and *SpiC^−/−^* mice infected with *P. chabaudi* for 20 days. Days depicted in blue and orange represent a 1.5-fold decrease or increase, respectively, in frequency in *SpiC^−/−^* mice compared to *SpiC^+/−^* mice; red asterisks represent a significant difference over the entire infection course (Wilcoxon matched pairs signed rank test, α = 0.05).
(PDF)

Table S1 Genes induced in whole blood by *P. chabaudi* at 24 h post-infection.
(XLSX)

Appendix S1 Supporting experimental procedures.
(PDF)
