# Supplementary figures and images for: Splenic Red Pulp Macrophages Produce Type I Interferons as Early Sentinels of Malaria Infection but Are Dispensable for Control
Source: PLoS One. 2012 Oct 29;7(10):e48126. doi: 10.1371/journal.pone.0048126 (PMC3483282; doi:10.1371/journal.pone.0048126)

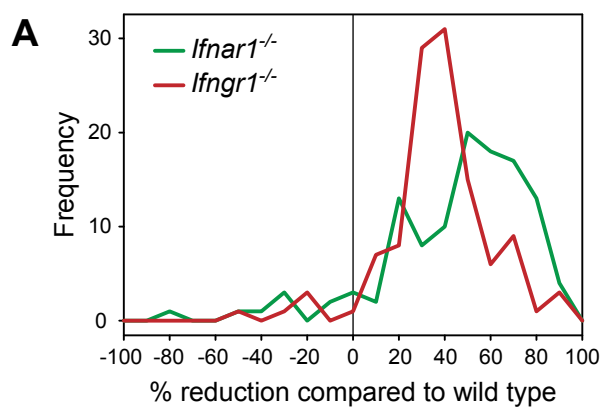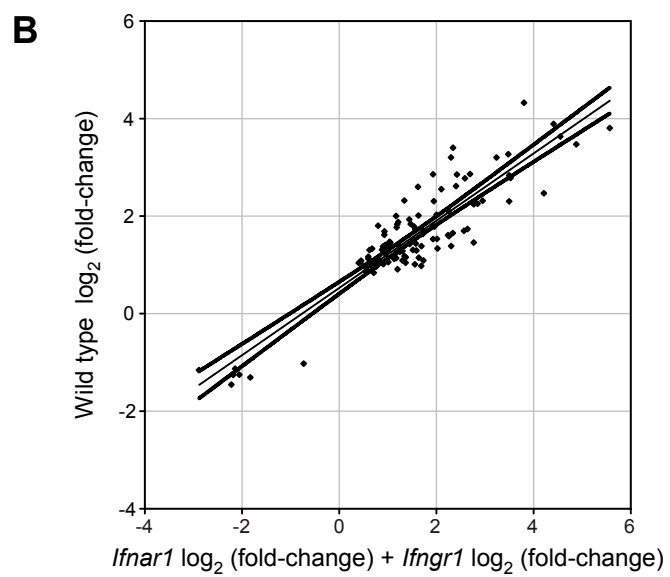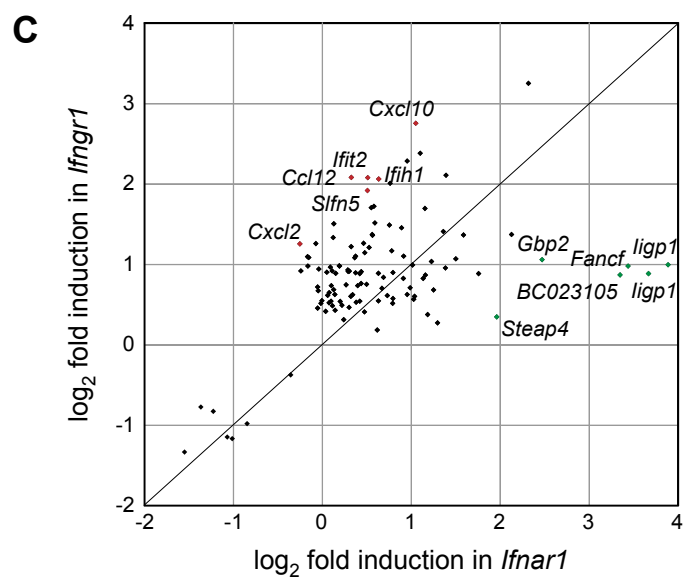

Supplement: Figure S1 — Redundancy and specificty in interferon signaling. (A) The distribution of percent reduction in fold-induction for individual ISG in IFN receptor knockout mice. (B) The sum of the average magnitudes of T1IFN and IFNG gene induction amount to more than the whole observed in wild type mice, indicating redundancy in gene expression. Each point represents a different probe, and lines represent the linear regression and 95% confidence interval. (C) A subset of ISG exhibit preferential induction by either T1IFN or IFNG. The log2 fold induction of the 117 early response genes is plotted for Ifnar1−/− and Ifngr1−/− mice to identify preferentially induced genes. Residuals from identity (x = y) were calculated, and an arbitrary cutoff of 1.4 was chosen to highlight the most distant genes (i.e. the most preferentially induced genes). Green points represent genes preferentially induced by IFNG, and red points denote genes preferentially induced by T1IFN. (PDF) [file pone.0048126.s001.pdf]

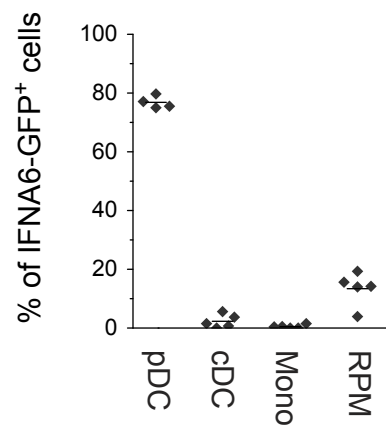

Supplement: Figure S2 — Induction of Ifna6-Gfp expression in splenic leukocytes by P. chabaudi . GFP+ events were analyzed for lineage markers 24 h after infection as in Fig. 4. (PDF) [file pone.0048126.s002.pdf]

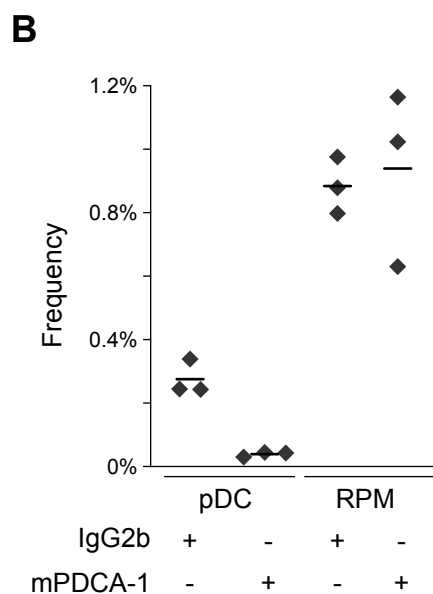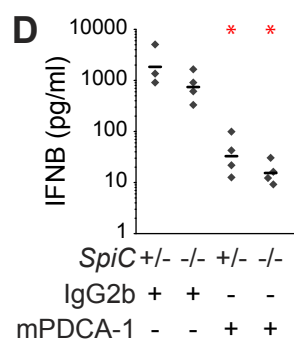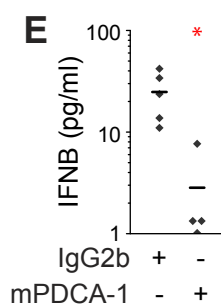

Supplement: Figure S3 — Both pDCs and RPMs are required for full T1IFN production during P. chabaudi infection. (A) Live singlet cells were subjected to lineage marker analysis for myeloid populations, demonstrating that SpiC−/− mice exhibit reduced RPM frequency compared to SpiC+/− animals, but otherwise have intact splenic macrophage and dendritic cell populations. (B) Treatment of C57BL/6 mice with mPDCA-1 antibody depletes splenic pDC populations but does not affect red pulp macrophages. Data represents frequencies measured after 18 h depletion plus 24 h infection with P. chabaudi. (C) Plasma IFNB levels are diminished in 129Sv SpiC−/− compared to 129Sv SpiC+/− mice. (D) Deficiencies in RPM and pDCs diminish the plasma IFNB response to P. chabaudi in 129Sv SpiC−/− mice. (E) Depletion of pDCs in C57BL/6 mice decreases the plasma IFNB response to P. chabaudi. Asterisks represent p<0.05 in a two-tailed t-test assuming unequal variances compared with intact controls. (PDF) [file pone.0048126.s003.pdf]

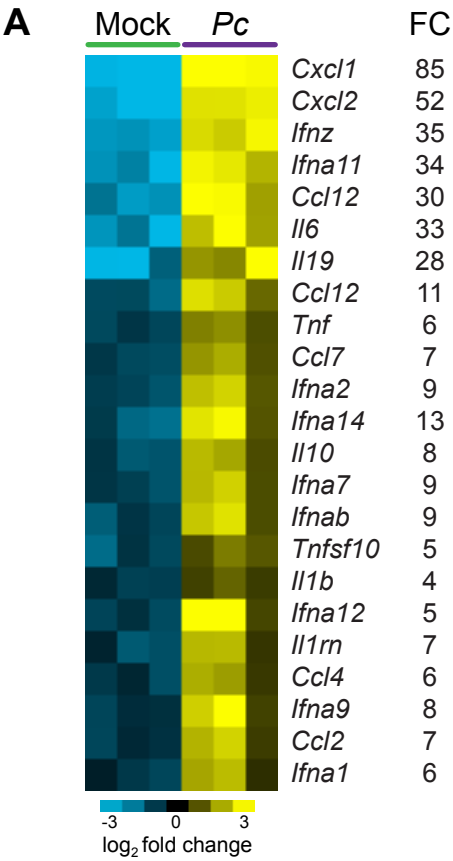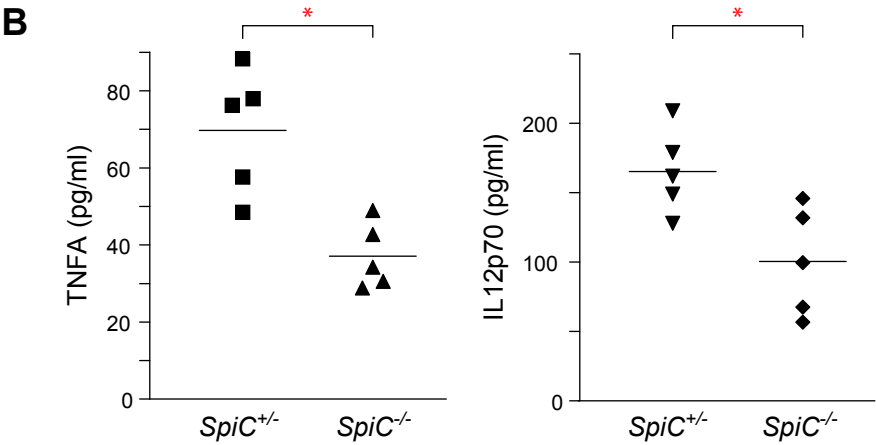

Supplement: Figure S4 — RPMs induce expression of Ifna and other cytokines and chemokines in response to P. chabaudi infection. (A) RNA was harvested from FACS-isolated RPMs from mock- or P. chabaudi-infected C57BL/6 animals, amplified, and hybridized to microarrays. A representative set of cytokines and chemokines induced upon infection are shown with fold change in transcript abundance. (B) Plasma cytokines of 129Sv SpiC+/− and SpiC−/− mice infected for 24 h with P. chabaudi were measured using Milliplex analysis (Millipore) on a MagPix instrument (Luminex). Differences between SpiC+/− and SpiC−/− mice are significant by a two-tailed t-test assuming unequal variances (α = 0.05; red asterisks). (PDF) [file pone.0048126.s004.pdf]

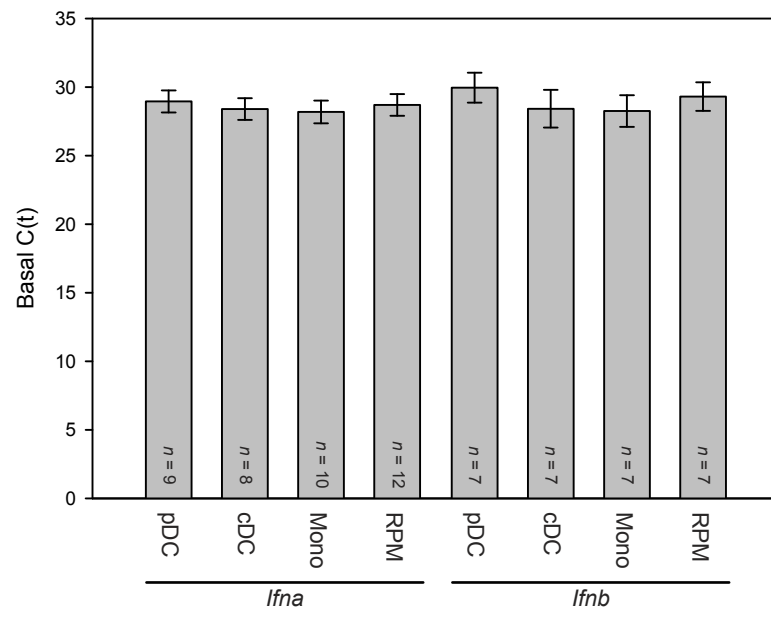

Supplement: Figure S5 — Basal C(t) values for leukocyte subsets. FACS-sorted populations from mock-infected animals were subjected to qRT-PCR for T1IFN transcripts. The data were aggregated from 4 independent experiments, with means and 95% confidence intervals represented. No significant differences were observed for any populations. (PDF) [file pone.0048126.s005.pdf]

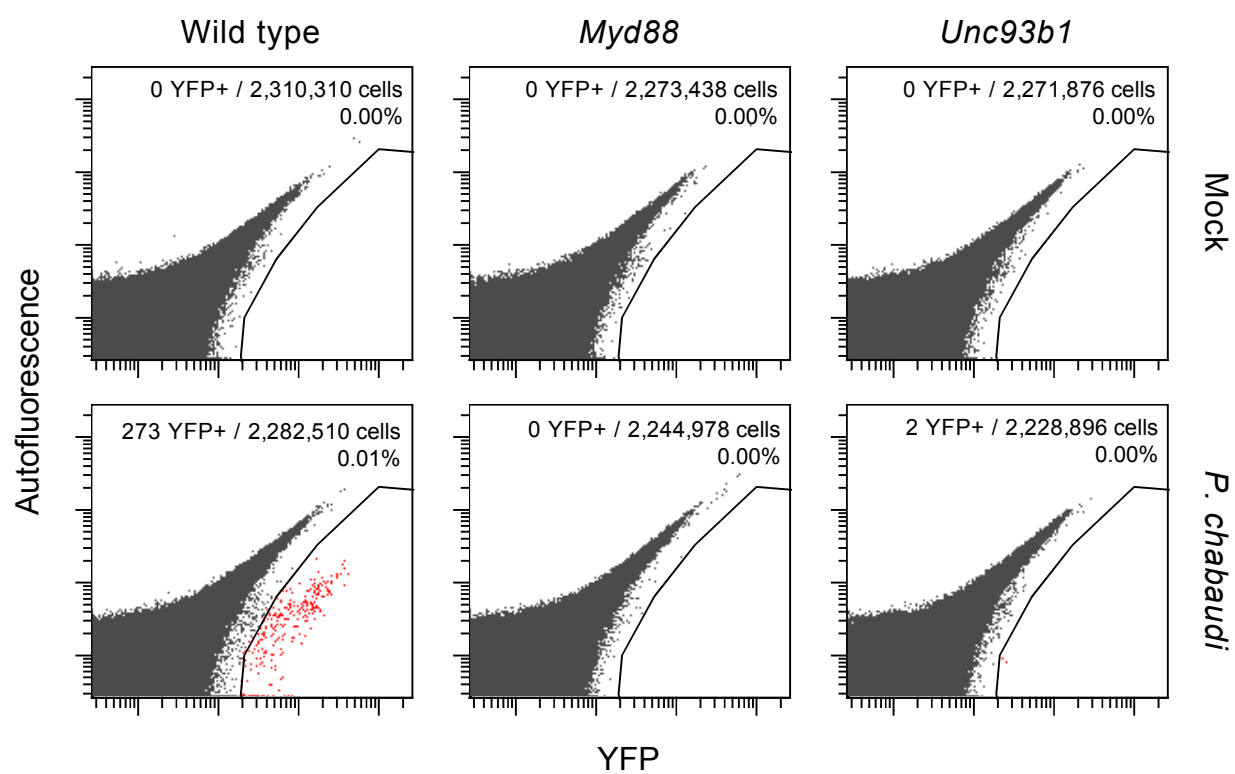

Supplement: Figure S6 — MYD88 is required for Ifnb-Yfp induction. Mice were inoculated with 106 infected erythrocytes or mock-infected with uninfected erythrocytes and spleens were harvested and processed for flow cytometry 24 h later. (PDF) [file pone.0048126.s006.pdf]

**A**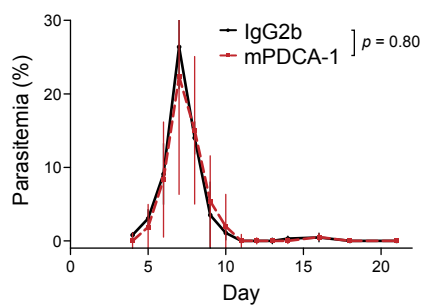**B**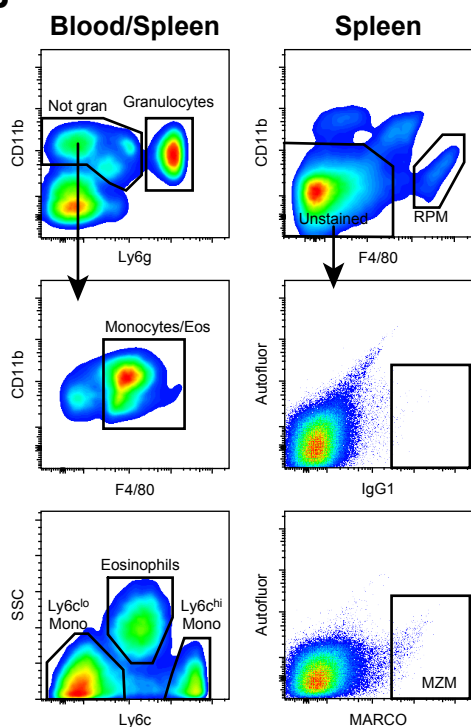**C**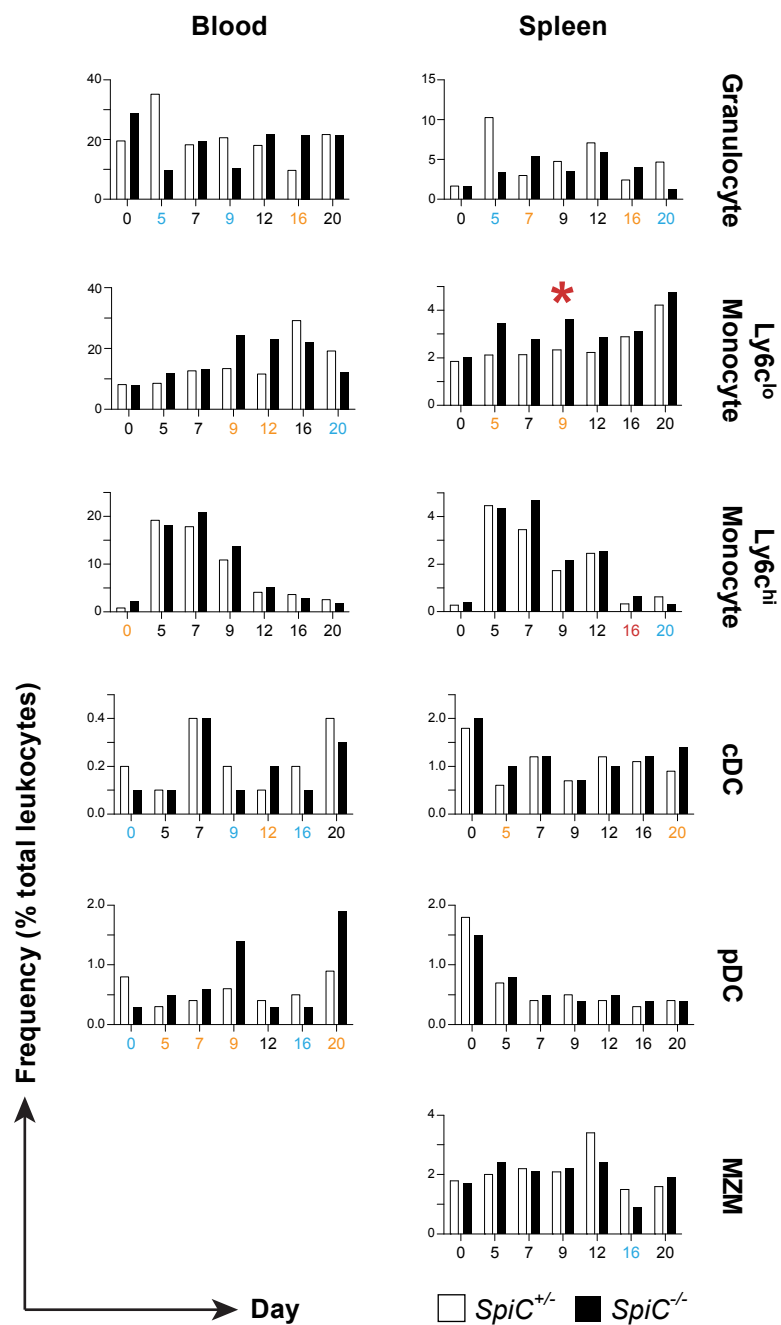

Supplement: Figure S7 — pDC and RPM are both dispensable for the control of P. chabaudi parasitemia. (A) C57BL/6 mice were intraperitoneally infected with 106 parasites. On day 4 post-infection, 500 µg of anti-mPDCA-1 antibody (Miltenyi Biotec) or IgG2b isotype control antibody (LTF2, UCSF hybridoma core) was administered intraperitoneally. Parasitemias are presented as geometric means with standard deviations and Mann-Whitney p-value. (B) Gating strategy for identification of myeloid populations in blood and spleen. Live singlet cells (not shown) were subjected to lineage marker analysis. MZM = marginal zone macrophages. (C) Myeloid population frequencies in blood and spleens of 129Sv SpiC+/− and SpiC−/− mice infected with P. chabaudi for 20 days. Days depicted in blue and orange represent a 1.5-fold decrease or increase, respectively, in frequency in SpiC−/− mice compared to SpiC+/− mice; red asterisks represent a significant difference over the entire infection course (Wilcoxon matched pairs signed rank test, α = 0.05). (PDF) [file pone.0048126.s007.pdf]
