# Supplementary material for: Splenic Red Pulp Macrophages Produce Type I Interferons as Early Sentinels of Malaria Infection but Are Dispensable for Control
Source: PLoS One. 2012 Oct 29;7(10):e48126. doi: 10.1371/journal.pone.0048126 (PMC3483282; doi:10.1371/journal.pone.0048126)
Supplement: Appendix S1 — Supporting experimental procedures. (PDF) [file pone.0048126.s009.pdf]

Splenic Red Pulp Macrophages Produce Type I Interferons as Early Sentinels of Malaria Infection but are Dispensable for Control

Charles C. Kim[a], Christopher S. Nelson[b], Emily B. Wilson[b], Baidong Hou[c,e], Anthony L. DeFranco[c], Joseph L. DeRisi[b,d,1]

[a]Division of Experimental Medicine, Department of Medicine
[b]Department of Biochemistry and Biophysics, and
[c]Department of Microbiology and Immunology,
[d]Howard Hughes Medical Institute
University of California, San Francisco, CA, 94143, USA
[e]Institute of Biophysics, Chinese Academy of Sciences, Beijing 100101, China

Supplemental Experimental Procedures

Microarray hybridization. All whole blood samples were hybridized against a reference of pooled samples supplemented with amplified Universal Mouse Reference RNA (Stratagene, 740100). Red pulp macrophage hybridizations were performed against a common aRNA pool from amplified red pulp macrophage samples. In all experiments, Cy5 was used to label the experimental sample and Cy3 was used to label the reference. Sample and reference amplified RNA were combined and mixed with hybridization solution (polyA, yeast tRNA, HEPES, SSC, SDS), boiled, and hybridized to mouse whole-genome microarrays for 15-17 h at 65°C under MAUI AO mixing chambers. The MEEBO microarray probe set was utilized due to its high genome coverage and constitutive exonic design (1).

Microarray Analysis. Image data were extracted in Genepix 6 (Molecular Devices) and normalized and filtered in Acuity 4 (Molecular Devices). Data were ratio-normalized, and control and poor quality features (as determined by both visual examination and application of quantitative filters for saturation, feature diameter, and variance) were removed. Data were further filtered for spots that did not exhibit signal in any of the sample channels (based on percentage of pixels in the feature above background and feature intensity) and for excessive missing data. The ratio of medians of the remaining data was transformed to $\log_2$ space, median centered by array, and median centered by gene.

The normalized and filtered data were analyzed for differential gene expression by hierarchical clustering using Xcluster (2) and statistical analysis in Significance Analysis of Microarrays (SAM) (3). All SAM analyses were conducted as two-class unpaired analyses using a *t*-statistic, K-nearest neighbor imputation of missing values, and cutoffs set at a 1% false discovery rate. Microarray data were visualized in Java Treeview (4). Significant gene sets were subject to functional analysis using gene ontology analysis with DAVID (5, 6).

References

1. Verdugo RA, Medrano JF (2006) Comparison of gene coverage of mouse oligonucleotide microarray platforms. *BMC Genomics* 7:58.

2. Gollub J, Sherlock G (2006) Clustering microarray data. *Meth. Enzymol* 411:194-213.

3. Tusher VG, Tibshirani R, Chu G (2001) Significance analysis of microarrays applied to the ionizing radiation response. *Proc. Natl. Acad. Sci. U.S.A* 98:5116-5121.

4. Saldanha AJ (2004) Java Treeview--extensible visualization of microarray data. *Bioinformatics* 20:3246-3248.

5. Huang DW, Sherman BT, Lempicki RA (2009) Bioinformatics enrichment tools: paths toward the comprehensive functional analysis of large gene lists. *Nucleic Acids Res* 37:1-13.

6. Huang DW, Sherman BT, Lempicki RA (2009) Systematic and integrative analysis of large gene lists using DAVID bioinformatics resources. *Nat Protoc* 4:44-57.
